# Loss of the endothelial glycocalyx is associated with increased E-selectin mediated adhesion of lung tumour cells to the brain microvascular endothelium

**DOI:** 10.1186/s13046-015-0223-9

**Published:** 2015-09-25

**Authors:** Srijana Rai, Zaynab Nejadhamzeeigilani, Nicholas J. Gutowski, Jacqueline L. Whatmore

**Affiliations:** Institute of Biomedical and Clinical Science, University of Exeter Medical School, St. Luke’s campus, Exeter, EX1 2LU UK; Royal Devon and Exeter NHS Foundation Trust, Barrack road, Exeter, EX2 5DW UK

**Keywords:** Adhesion, Endothelial cells, E-selectin, Glycocalyx, Lung cancer

## Abstract

**Background:**

Arrest of metastasising lung cancer cells to the brain microvasculature maybe mediated by interactions between ligands on circulating tumour cells and endothelial E-selectin adhesion molecules; a process likely to be regulated by the endothelial glycocalyx. Using human cerebral microvascular endothelial cells and non-small cell lung cancer (NSCLC) cell lines, we describe how factors secreted by NSCLC cells *i.e.* cystatin C, cathepsin L, insulin-like growth factor-binding protein 7 (IGFBP7), vascular endothelial growth factor (VEGF) and tumour necrosis factor-alpha (TNF-α), damage the glycocalyx and enhance initial contacts between lung tumour and cerebral endothelial cells.

**Methods:**

Endothelial cells were treated with tumour secreted-proteins or lung tumour conditioned medium (CM). Surface levels of E-selectin were quantified by ELISA. Adhesion of A549 and SK-MES-1 cells was examined under flow conditions (1 dyne/cm^2^). Alterations in the endothelial glycocalyx were quantified by binding of fluorescein isothiocyanate-linked wheat germ agglutinin (WGA-FITC).

**Results:**

A549 and SK-MES-1 CM and secreted-proteins significantly enhanced endothelial surface E-selectin levels after 30 min and 4 h and tumour cell adhesion after 30 min, 4 and 24 h. Both coincided with significant glycocalyx degradation; A549 and SK-MES-1 CM removing 55 ± 12 % and 58 ± 18.7 % of WGA-FITC binding, respectively. Inhibition of E-selectin binding by monoclonal anti-E-selectin antibody completely attenuated tumour cell adhesion.

**Conclusion:**

These data suggest that metastasising lung cancer cells facilitate their own adhesion to the brain endothelium by secreting factors that damage the endothelial glycocalyx, resulting in exposure of the previously shielded adhesion molecules and engagement of the E-selectin-mediated adhesion axis.

**Electronic supplementary material:**

The online version of this article (doi:10.1186/s13046-015-0223-9) contains supplementary material, which is available to authorized users.

## Introduction

Brain metastases arising from primary lung cancer contribute significantly to the morbidity and mortality of the disease. These tumours present a challenging clinical scenario since they are generally resistant to conventional chemotherapy due to the inability of these drugs to cross the blood–brain barrier (BBB) [1]. Insights into the molecular mechanisms of the metastatic process will aid development of more effective therapeutic strategies.

In order to form metastases, tumour cells must successfully complete a sequence of key, inter-related steps initiated by extensive proliferation of the primary tumour cells and their invasion of the surrounding extracellular matrix (ECM). These malignant cells then dissociate from the primary site, intravasate the circulation and form tumour microemboli, which eventually arrest along the microvasculature of a target organ. Tumour cells attach to the endothelium, extravasate and ultimately colonize to form secondary metastatic lesions. Although, the exact mechanisms of tumour cell adhesion in the vasculature of specific organs remain unknown, parallels have been drawn with the leukocyte-endothelial cell (EC) interactions that occur during inflammation. Here, initial leukocyte ‘tethering’ and ‘rolling’ is primarily mediated by endothelial surface proteins from the selectin family (E- and P- selectin) whereas stronger bonds resulting in leukocyte arrest, firm adhesion and finally transmigration are determined by members of the endothelial immunoglobulin superfamily [intercellular adhesion molecule-1 (ICAM-1) and vascular cell adhesion molecule-1 (VCAM-1)]. These same adhesion molecules may be involved in metastatic tumour cell adhesion [2, 3]. Several studies [4, 5] have shown that altered E-selectin expression on activated endothelial cells greatly influences metastasis formation and subsequent colonization, and increased circulating E-selectin levels have been associated with metastasis of both breast and colon carcinoma cells to the liver [6, 7]. Data on the role of endothelial adhesion molecules in lung tumour metastasis to the brain are very limited; however Soto *et al.* did recently report a role for VCAM-1 and activated leukocyte cell adhesion molecule (ALCAM) in the early stages of brain metastasis in a mouse model [8].

It is now becoming recognised that a major physiological regulator of adhesion of circulating cells to the endothelium is the endothelial surface layer (glycocalyx); a dense intraluminal layer of transmembrane and membrane-bound molecules [9]. It is physically linked to the endothelium through key ‘backbone’ proteoglycans and glycoproteins, which together form a mesh into which both endothelium- and plasma-derived soluble molecules are incorporated [10]. The glycocalyx is a dynamic structure maintained by a tightly controlled equilibrium between degradation and synthesis of the principal components.

If lung tumour cells utilise the cerebral endothelial adhesion molecules to initially adhere to the brain microvasculature during metastasis, then the glycocalyx may play a critical role in this process by sterically influencing the ligand-receptor binding dynamics. In health, the EC adhesion molecules are embedded within the glycocalyx layer since glycocalyx thickness has been reported up to 1 μm; considerably larger than the 10 nm span of EC adhesion molecules and receptors [11]. Thus, endothelial adhesion molecules are exposed by loss of the glycocalyx. This raises the intriguing possibility that circulating lung tumour microemboli arrested in the cerebral microcirculation have a metastatic potential that enables them to secrete factors that locally degrade the glycocalyx; enhancing endothelial adhesion molecule exposure and facilitating their own adhesion and ultimately transmigration. Thus the objective of this study was to identify key pro-metastatic factors secreted by lung tumour cells and to investigate the effect of these factors on cerebral EC adhesion molecule exposure, glycocalyx integrity and binding of lung tumour cells.

Here, we report for the first time that human non-small cell lung cancer (NSCLC) cells (A549; adenocarcinoma and SK-MES-1; squamous cell carcinoma) secrete factors that enhance human cerebral endothelial adhesion molecule exposure and increase adhesion of tumour cells to the cerebral endothelium. Importantly, we identify E-selectin as the key adhesion molecule involved in the initial tethering of the lung tumour cells and for the first time demonstrate that loss of integrity of the cerebral endothelial glycocalyx enhances exposure of E-selectin and tumour cell adhesion. Our results highlight a potentially important mechanism by which malignant lung tumour cells can enhance their own metastatic potential; raising the possibility that interventions designed to maintain glycocalyx integrity may have important clinical benefit in reducing brain metastases in primary lung tumour patients.

## Materials and methods

### Cell culture and preparation of tumour conditioned-medium

A transformed human cerebral microvascular endothelial cell line, hCMEC/D3, was kindly provided by Dr. Pierre-Olivier Couraud, Institut Cochin, INSERM U1016, France. This cell line has been fully characterized by Dr Couraud and shown to express a range of endothelial and BBB-specific markers [12]. Cells were cultured and grown to confluence in rat tail collagen type I coated tissue culture flasks at 37 °C under 5 % CO_2_ atmosphere using Endothelial Cell Basal MV2 Medium (C-22221, PromoCell, Germany) supplemented with MV2 supplement pack (C-39221, PromoCell, Germany). For experiments, hCMEC/D3 cells were between passage 25 and 35.

Human NSCLC cell lines, A549 and SK-MES-1, were purchased from the European Collection of Cell Cultures (ECACC; Salisbury, UK) and cultured in Dulbecco’s Modified Eagle Medium (DMEM, Lonza, Slough, UK) supplemented with L-glutamine (2 mM), gentamicin (50 μg/ml) and 10 % (v/v) FBS (Sigma Aldrich, Gillingham, UK).

For collection of lung tumour conditioned-medium (CM), A549 and SK-MES-1 cells at 80 % to 90 % confluence were washed with PBS (x3) and incubated with chemically defined DMEM-BS medium [13] (1 ml of medium per 7.5 cm^2^ of growth area) for 24 h at 37 °C. Media were then centrifuged (600 *g*, 10 min, 4 °C) and stored at −80 °C for further experiments.

### Mass spectrometry analysis and ELISA

ELISA and LC-MALDI TOF/TOF mass spectrometry (MS) were carried out to screen the secretome (as CM) of A549 and SK-MES-1 cells (see Additional file 1 for experimental details). Analyses were also carried out on DMEM-BS as a control. DMEM-BS is a serum-free chemically defined medium specially formulated by Bottenstein & Sato from DMEM [13]. Since the formulation is known, any additional factors secreted into CM could be easily identified. Significant results were determined by selecting proteins that matched with two or more peptides with total ion CIs of >95 %, followed by a literature search to determine proteins with a possible role in metastasis. The following tumour secreted metastasis-related proteins were identified from the available literature: tumour necrosis factor-alpha (TNF-α) [14], cathepsin L (CL) [15], cystatin C (CC) [16], vascular endothelial growth factor (VEGF) [17] and insulin-like growth factor binding protein-7 (IGFBP-7) [18].

Secreted levels of TNF-α, CL, CC, VEGF-A and IGFBP-7 in the A549 and SK-MES-1 CM were measured by ELISA (TNF-α and CC: Bender MedSystems, Vienna, Austria; CL: R&D systems, Abingdon, UK; VEGF-A: PeproTech®, London, UK) and IGFBP-7: in house ELISA previously described in [18].

### Treatment conditions

During the various investigations, with the exception of E-selectin ELISA, hCMEC/D3 cells were incubated with either complete lung tumour CM (A549 CM and SK-MES-1 CM) or individual tumour secreted-proteins (at concentrations detected during the previous ELISAs) – 80 ng/ml CC (Abcam, Cambridge, UK), 10 ng/ml CL (Sigma Aldrich, Gillingham, UK), 200 ng/ml IGFBP-7 (Abcam, Cambridge, UK), 0.2 ng/ml VEGF-A (R&D Systems, Abingdon, UK) and 160 pg/ml TNF-α (Enzo Life sciences, Exeter, UK). All of the aforementioned solutions were prepared in DMEM-BS, and control cells received DMEM-BS only.

### Cell surface based ELISA (E- and P-selectin)

Surface levels of E- and P-selectin on human cerebral endothelial cells (ECs) were assessed using a semi-quantitative cell based ELISA as previously described in [19]. Cells were cultured in collagen I coated 96-well plates; once a monolayer had formed they were serum starved overnight and treated with increasing concentrations of identified tumour secreted-proteins (in DMEM-BS) for 4 h; CC – 8, 80 and 800 ng/ml, CL – 1, 10 and 100 ng/ml, IGFBP-7 – 100, 300 and 900 ng/ml, VEGF-A – 0.2, 10 and 20 ng/ml and TNF-α – 100, 500 and 2500 pg/ml and/or lung tumour CM. Exposed selectin molecule was recognised by an anti-E- or P-selectin antibody and all subsequent ELISA incubation and washing steps are described in Additional file 1.

### Laminar flow adhesion assay

The adhesion of lung cancer cells to the brain endothelium was assessed using an *in vitro* flow model, where treated hCMEC/D3 monolayers in gas-permeable flow chambers were perfused with a suspension of lung tumour cells. ECs were cultured and treated as required in collagen I coated flow chambers (ibidi μ-slides I and VI^0.4^, Thistle Scientific, Glasgow UK). After 4 and 24 h, A549 or SK-MES-1 cells (1 x 10^6^ cells/mL) stained with 0.5 μM calcein acetoxymethyl ester (Calcein AM; Life Technologies Ltd, Paisley UK) were flowed over the monolayers at a flow rate calculated to provide a shear stress of 1dyne/cm^2^, *i.e.* low shear stress comparable to that found in post-capillary venules where the majority of adhesion occurs, for 10 min at 37 °C [20]. To remove any non-adherent tumour cells, the slides were rinsed with PBS for 2.5 min at 37 °C before being fixed with 4 % paraformaldehyde. The flow slides were then examined under a Nikon Eclipse TS100 fluorescence microscope and the number of adherent A549 or SK-MES-1 cells in 10 random fields of view were counted, concentrating on central areas rather than borders.

For the E-selectin inhibition experiments, endothelial monolayers were treated for 24 h with CM and secreted-proteins and then exposed to monoclonal anti-E-selectin antibody (5 μg/ml, clone 1.2B6, Sigma Aldrich, Gillingham, UK) for 30 min prior to perfusion with calcein AM stained A549 and SK-MES-1 cell suspensions (as described above). The optimum conditions for anti-E-selectin treatment were determined previously through time- and concentration-dependent inhibitory experiments.

### Flow cytometry analysis of adhesion molecule counter ligands/receptors

Expression of adhesion molecule counter ligands and receptors on A549 and SK-MES-1 lung tumour cells were investigated by flow cytometry (FC); specifically the E-selectin ligand sialyl Lewis X (sLeX), ICAM-1 receptors, lymphocyte function-associated antigen 1 (LFA-1α/CD11a) and macrophage-1 (Mac-1α/CD11b) and VCAM-1 receptor, very late antigen-4 (VLA-4α/CD49d). See Additional file 1 for experimental details.

### Cell based fluorescence assay

A cell-based fluorescence (CBF) assay, as previously described by Singh and colleagues [21], was performed to quantify changes within the glycocalyx using fluorescein isothiocyanate-labelled wheat germ agglutinin (WGA-FITC), which binds to *N*-acetyl neuraminic acid and *N*-acetyl glucosamine residues of proteoglycans and glycoproteins present in the glycocalyx.

Lectin-binding (as WGA-FITC binding) was also observed (as previously described [22]), before and after 1 h treatment with lung tumour CM, with a confocal laser scanning microscope (Leica DMi8 TCS SP8 Confocal, Leica Microsystems, Germany). The images (1024 x 1024 pixels) were acquired with a 10x tube lens and processed using the Leica LAS X Core software.

### Glycocalyx constituents ELISA – Hyaluronic acid and Syndecan-1

Hyaluronic acid (HA) and syndecan-1 (CD138) levels in CM-treated endothelial supernates were measured using the R&D Systems (Abingdon, UK) Quantikine Hyaluronan Immunoassay kit (DHYAL0) and the Syndecan-1 Human ELISA kit (ab46506) by Abcam (Cambridge, UK). See Additional file 1 for experimental details.

### Statistical analysis

Data are presented as mean ± standard deviation (SD) and statistical differences between control and treatment groups were assessed using Mann–Whitney non-parametric test. *P*-value ≤ 0.05 is considered statistically significant.

## Results

### Lung cancer cells secrete a range of factors with a potential role in mediating brain metastasis

**Fig. 1:**
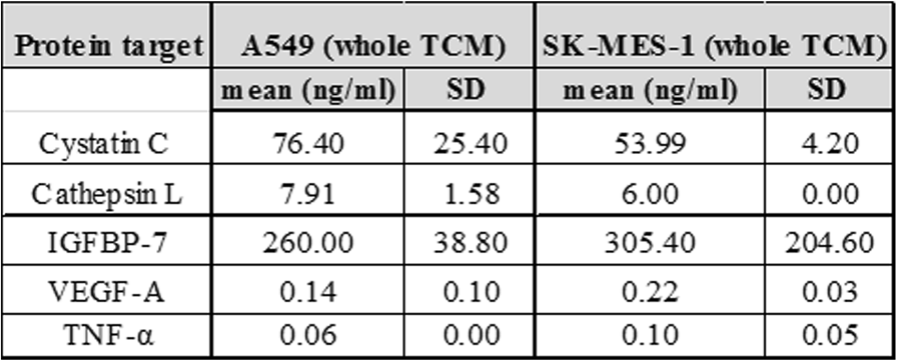
Potential prometastatic proteins were identified and quantified in human NSCLC cell secretome. ELISA analysis of CC, CL, IGFBP-7, TNF-α and VEGFA concentration in A549 and SK-MES-1 CM. All experiments were carried out in duplicate on three separate samples

Initially, MS was used to screen the secretome (as CM) of A549 and SK-MES-1 cells for the presence of metastasis-related proteins. As expected both cell types secreted a large range of proteins not present in the DMEM-BS control (see tables S1, S2 and S3 in Additional file 2). However, only the following three proteins were identified to have a possible role in metastasis and investigated further: CC, CL, and IGFBP-7. Surprisingly, no growth factors or cytokines were detected using this approach, presumably due to limitations of MS in identifying proteins in complex mixtures, such as CM, and instrument limitation. Thus, ELISAs were carried out to quantify the secreted levels of the proteins identified above and to investigate whether the tumour cells secreted other known pro-angiogenic factors including VEGF-A and TNF-α. Figure 1 shows the concentrations of VEGF-A, TNF-α, CC, CL, and IGFBP-7 in CM from both cancer cell lines. The concentrations of measured proteins did not vary greatly between A549 and SK-MES-1 CM.

### Lung tumour secreted-proteins induce increased exposure of selectins on the surface of human cerebral ECs

**Fig. 2:**
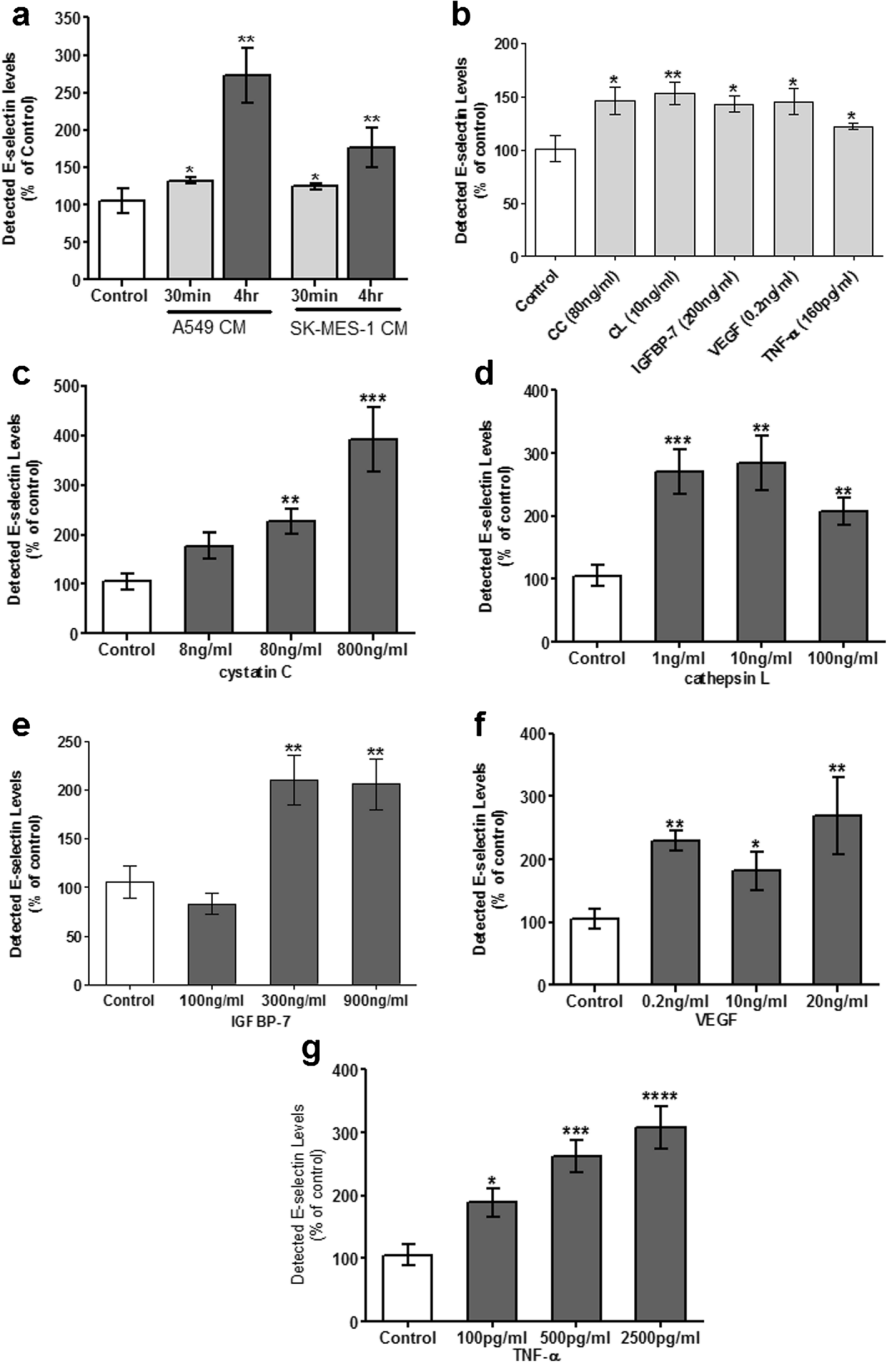
Increased levels of E-selectin detected on the surface of human cerebral microvascular endothelial cells in response to lung tumour CM and secreted-proteins. Confluent hCMEC/D3 cells were stimulated for 4 h with A549 and SK-MES-1 CM (**a**) along with CC (**c**), CL (**d**), IGFBP-7 (**e**), VEGF (**f**) and TNF-α (**g**). E-selectin levels from 3 separate experiments, carried out in quadruplets, were statistically analysed (n ≥ 12, mean ± SD, *p ≤ 0.05 and **p ≤ 0.01, ***p ≤ 0.005 and ****p ≤ 0.0001). Enhanced E-selectin was also detected within 30 min of CM (**a**) and secreted-proteins (**b**) exposure (*p ≤ 0.05 and **p ≤ 0.01, *n* = 6)

Metastasis critically requires adhesion of the circulating tumour cells (CTCs) to the endothelium of secondary organs, possibly utilising endothelial selectins. Thus, the influence of the lung tumour secreted proteins (at a concentration range based on protein levels measured in CM) on cerebral EC E- and P-selectin surface levels was investigated. Enhanced E-selectin surface expression typically requires *de novo* synthesis usually triggered by an inflammatory stimulus and peaks approximately 4–6 h after activation. After 4 h, all tumour secreted-proteins induced a significant increase in surface E-selectin at either higher concentrations tested (CC, IGFBP-7) or over the whole concentration range (CL, VEGF-A, TNF-α) (Fig. 2). Based on the obtained data and the protein concentrations measured in CM, subsequent studies used only one concentration of each protein: CC 80 ng/ml; CL 10 ng/ml; IGFBP-7 200 ng/ml; VEGF-A 0.2 ng/ml and TNF-α 160 pg/ml. At these concentrations the proteins significantly increased detected endothelial E-selectin levels to 226 ± 73.1 %, 285 ± 129.2 %, 210 ± 70.9 %, 229 ± 45.1 % and 189 ± 59.1 % compared to control (100 %) (*p* ≤ 0.01).

Cerebral ECs exposed to both A549 and SK-MES-1 CM also demonstrated enhanced levels of surface E-selectin at 4 h (272 ± 96.1 % and 177 ± 70.4 % respectively). Interestingly, a significant increase was detected even after 30 min treatment (Fig. 2a) (132 ± 4.6 % and 125 ± 7.2 %) (*p* ≤ 0.05). This was also evident with the tumour-secreted proteins at their chosen concentrations (CC: 146 ± 42.3 %, CL: 153 ± 33.4 %, IGFBP-7: 143 ± 28.5 %, VEGF-A: 146 ± 41.0 % and TNF-α: 122 ± 11.2 %) (Fig. 2b).

Furthermore, at 30 min A549 CM also appeared to significantly enhance the exposure of P-selectin on the surface of hCMEC/D3 cells (128 ± 30.6 % v control 100 %) (*p* ≤ 0.037, n = 3). There was no increase in P-selectin levels detected with SK-MES-1 CM.

### Lung tumour secreted-proteins enhance adhesion of lung tumour cells to human cerebral microvascular endothelial cells

**Fig. 3:**
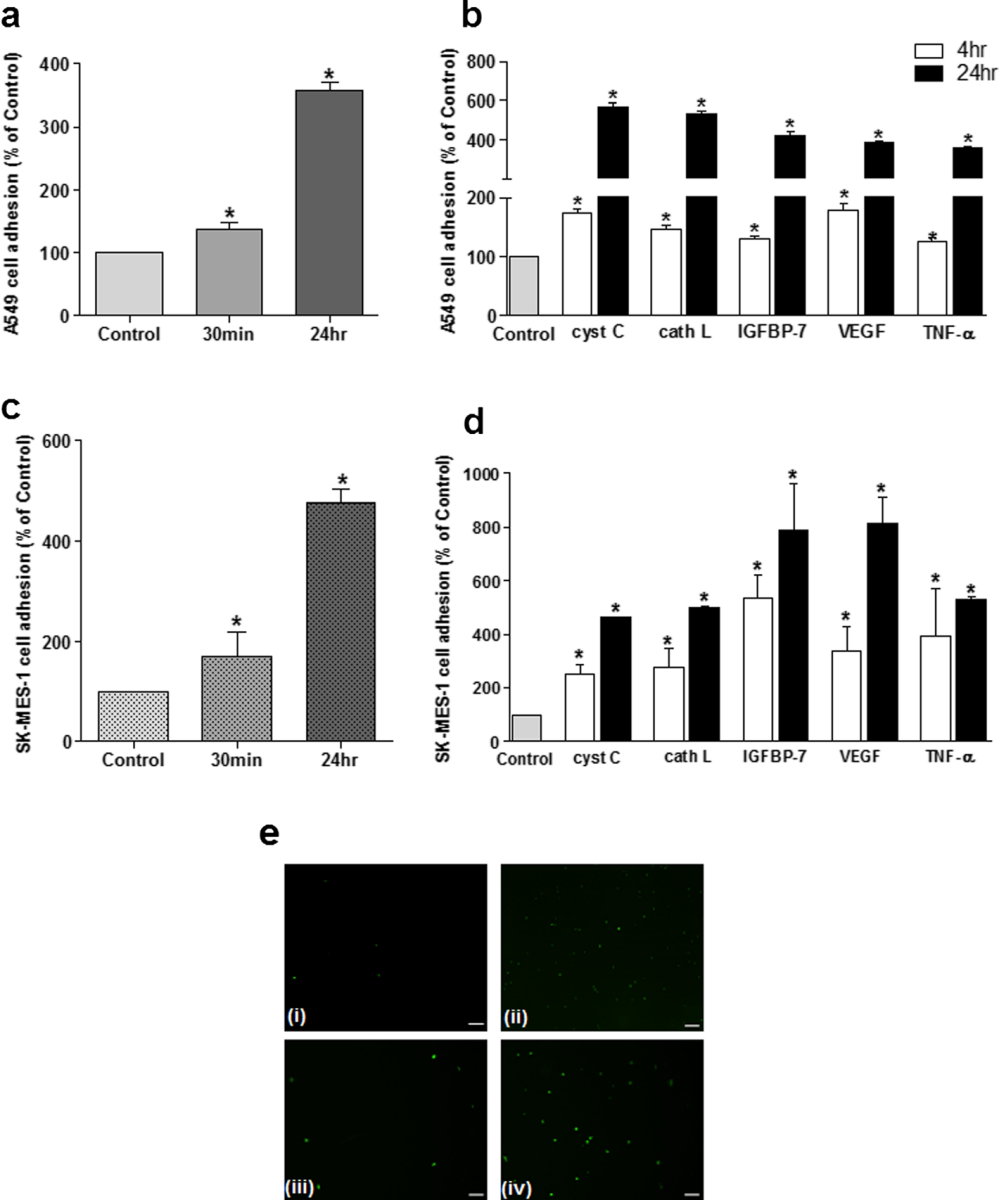
Lung tumour CM and secreted-proteins enhance adhesion of A549 and SK-MES-1 cells to human brain endothelial monolayers. Confluent hCMEC/D3 cells in μ-slides were exposed to A549 CM or SK-MES-1 CM for 30 min and 24 h prior to perfusion with calcein AM stained A549 (**a**) or SK-MES-1 cells (**c**). A549 and SK-MES-1 adhesion to hCMEC/D3 cells treated with 80 ng/ml CC, 10 ng/ml CL, 200 ng/ml IGFBP-7, 0.2 ng/ml VEGF and 160 pg/ml TNF-α at 4 and 24 h (**b**) and (**d**). Mean values from three separate experiments were statistically analysed. * *p* ≤ 0.05 versus control levels (100 %). **e** Representative images of adherent calcein-AM stained A549 [(*i*) and (*ii*)] and SK-MES-1 [(*iii*) and (*iv*)] cells to DMEM-BS [(*i*) and (*iii*)] and CL-treated [(*ii*) and (*iv*)] brain endothelial monolayers. Scale bar = 75 μm

The observation that A549 and SK-MES-1 lung tumour cells secrete factors that enhance surface levels of E-selectin led us to investigate whether this was reflected in altered adhesion of lung tumour cells (over 10 min) to similarly treated endothelial monolayers. Using a flow adhesion assay to mimic microvascular flow conditions, it was observed that adhesion of both lung tumour cell types to cerebral EC monolayers was significantly increased by previous exposure to relevant whole CM. With 24 h treatment, adhesion was enhanced to 358 ± 20.5 % (A549) and 476 ± 45.1 % (SK-MES-1 CM) compared to control levels (100 %) (*p* ≤ 0.05, Fig. 3a & c) Interestingly, a reduced, but still significant, increase in tumour cell adhesion was also observed after only 30 min of A549 and SK-MES-1 CM treatment; 137 ± 17.3 % and 170 ± 85.4 %, respectively. Treatment of cerebral ECs with all of the identified tumour secreted-proteins significantly increased adhesion of both lung tumour cell types to the monolayer. 24 h treatment with CC, CL, IGFBP-7, VEGF-A and TNF-α enhanced A549 cell attachment to 568 ± 40.5 %, 535 ± 22 %, 424 ± 36.1 %, 386 ± 18.6 % and 356 ± 18.8 %, respectively. Again, a reduced but still significant effect was observed at the shorter treatment time studied. Overall, SK-MES-1 cell adhesion was more pronounced in response to the secreted-proteins; at 24 h this rise was approximately 8-fold with IGFBP-7 and VEGF-A (788 ± 302.3 % and 817 ± 167.6 %). CC, CL and TNF-α also increased adhesion to 462 ± 4.6 %, 501 ± 10.7 % and 530 ± 15.6 %, respectively.

### Inhibiting E-selectin binding fully attenuated lung cancer cell adhesion to cerebral ECs

**Fig. 4:**
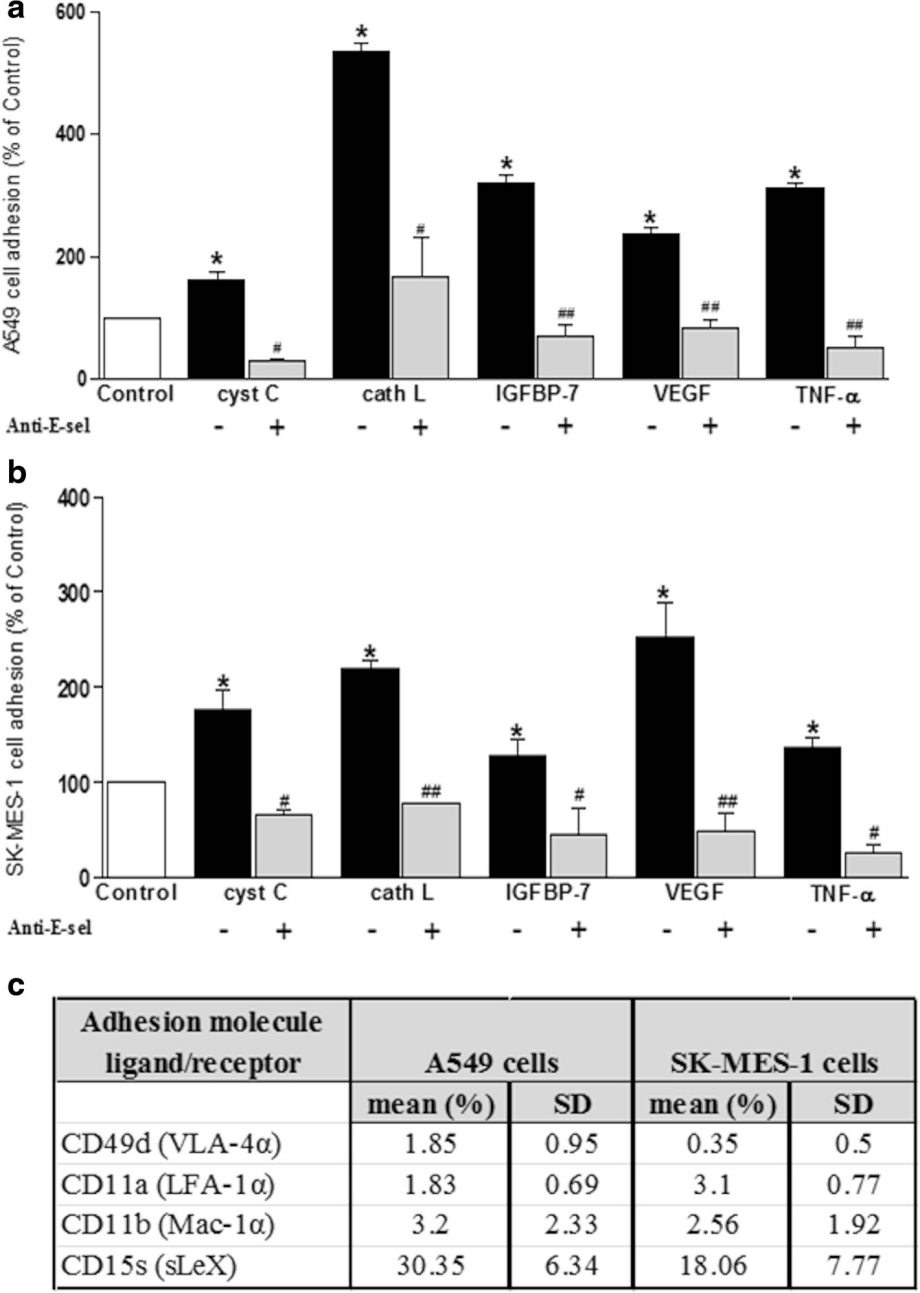
E-selectin inhibition attenuated adhesion of A549 and SKMES-1 lung cancer cells to human cerebral microvascular endothelial cells. Confluent hCMEC/D3 cells were treated with A549 CM (**a**), SK-MES-1 CM (**b**) and secreted-proteins for 24 h prior to exposing the relevant channels with anti-E-selectin antibody for 30mins. The bars represent cancer cell adhesion to brain endothelial monolayer calculated as a percentage of control obtained on 3 separate occasions. (* *p* ≤ 0.05 vs control, #*p* ≤ 0.05, ##*p* ≤ 0.01 vs anti-E-selectin). **c** FC analysis of adhesion molecule ligands and receptors, CD49d, CD11a, CD11b, PSGL-1 and sLeX, on lung tumour cells. The presented data was collected from 3 different experiments

These data suggest that lung cancer secreted-factors influence tumour cell adhesion to cerebral endothelial monolayers by inducing enhanced surface levels of E-selectin. To verify the role of E-selectin, blocking studies using a monoclonal antibody against E-selectin were performed. Treatment of the endothelial monolayer with anti-E-selectin antibody fully attenuated the increased adhesion of A549 and SK-MES-1 lung cancer cells (Fig. 4) induced by the tumour secreted-proteins. Isotype-matched control (mouse monoclonal IgG_1_) studies confirmed the specificity of the inhibition to the anti-e-selectin antibody (see Additional file 3: Figure S1).

Since these data strongly indicate a role for E-selectin in the adhesion of lung tumour cells to the cerebral endothelium, the expression of counter adhesion ligands and receptors on the tumour cells was investigated *i.e.* the E-selectin ligands sialyl Lewis X (sLeX) and P-selectin glycoprotein ligand-1 (PSGL-1), ICAM-1 receptors, lymphocyte function-associated antigen 1 (LFA-1α/CD11a) and macrophage-1 (Mac-1α/CD11b) and VCAM-1 receptor, very late antigen-4 (VLA-4α/CD49d). Both A549 and SK-MES-1 cells displayed relatively high levels of sialyl Lewis X compared with the other antigens investigated (Fig. 4c). Indeed, levels of VLA-4α, LFA-1α, PSGL-1 and Mac-1α were all very low (<5 %) on both cell types. Isotype-matched (IgM, κ) and positive controls corroborated these findings. The expression of VLA-4α, LFA-1α and Mac-1α was clearly evident (>30 %) on U937 cells and freshly isolated human monocytes, included as a positive control (see Additional file 4: Figure S2).

### The cerebral endothelial glycocalyx is significantly altered by lung tumour CM and secreted-proteins

E-selectin is transcriptionally regulated and increased surface expression is reported to take a minimum of 2 h. Thus, the observation that lung tumour secreted-factors significantly enhance both exposure of cerebral endothelium surface E-selectin and tumour /endothelial cell interactions within 30 min suggests that an additional, alternative mechanism contributes to regulation of tumour/endothelial interactions. The endothelial glycocalyx may influence cell adhesion interactions through steric effects on adhesion molecule/ligand binding dynamics. We therefore hypothesised that the lung tumour secretome could alter the integrity of the cerebral endothelial glycocalyx, leading to the increased exposure of previously ‘hidden’ E-selectin molecules and facilitating enhanced lung tumour cell adhesion.

**Fig. 5:**
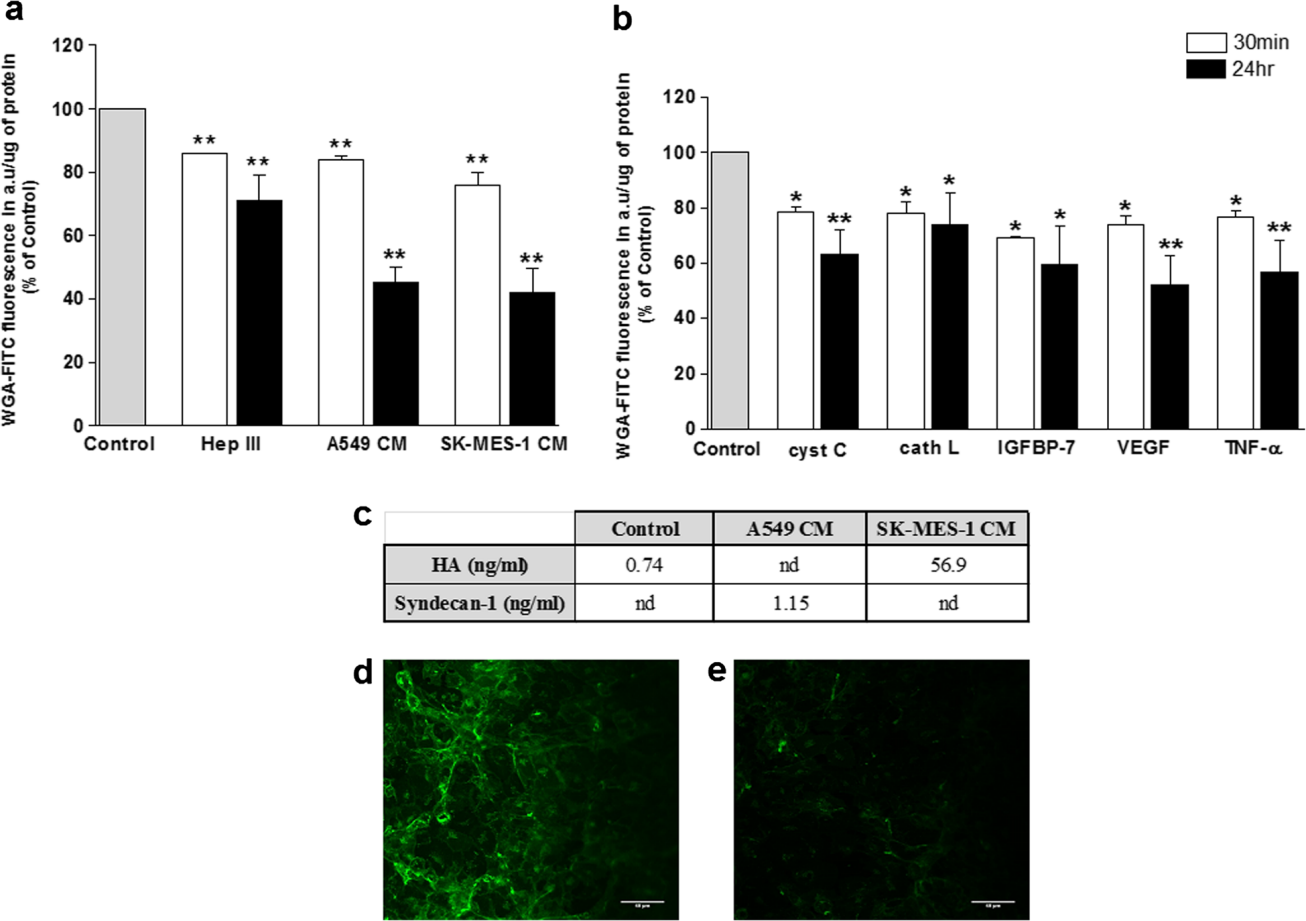
Exposure of lung tumour CM and secreted-proteins significantly altered the brain endothelial glycocalyx. **a** and **b** Integrity of the hCMEC/D3 glycocalyx was assessed by a CBF assay following exposure of ECs for 30 min or 24 h to A549 and SKMES-1 CM (**a**), or 80 ng/ml CC, 10 ng/ml CL, 200 ng/ml IGFBP-7, 0.2 ng/ml VEGF and 160 pg/ml TNF-α (**b**). Data from 6 independent experiments, carried out in sextuplicate, is presented as WGA-FITC fluorescence/μg protein and these values were calculated as a percentage of control. *p ≤ 0.05, **p ≤ 0.01 vs control level. **c** Hyaluronan (HA) and syndecan-1 levels were measured by ELISA in hCMEC/D3 growth medium following 30 min treatment with fresh DMEM-BS (control), A549 or SK-MES-1 CM. SK-MES-1 CM vs control = *p* = 0.007, *n* = 6. nd = not detected. **d** and **c** Representative confocal images of FITC-WGA stained hCMEC/D3 cells before (**d**) and (**e**) after 30 min exposure to SKMES-1 CM. Scale bar = 40 μM

A cell-based fluorescence assay was used to quantify changes within the glycocalyx. Significant reduction in glycocalyx staining of cerebral ECs occurred rapidly *i.e.* within 30 min of lung tumour CM treatment (A549 CM: 84 ± 1.7 % and SK-MES-1 CM: 76 ± 7.1 %, vs. control, 100 %, Fig. 5a, d, e), suggesting loss of glycocalyx integrity. An even greater reduction in glycocalyx staining was observed after 24 h to 45 ± 10.9 % and 42 ± 17.1 % after exposure to A549 and SK-MES-1 CM, respectively vs control (100 %). Interestingly, the effect of CM was more pronounced than that of the *F. heparinum* heparinase III enzyme (positive control, 71 ± 15.9 %), which exclusively cleaves heparan sulphate glycosaminoglycans within the glycocalyx (Fig. 5a). Treatment of the endothelial monolayer with CC, CL, IGFBP-7, VEGF-A and TNF-α also significantly altered the cerebral endothelial glycocalyx at both time points, with the greatest reduction in staining observed with 24 h VEGF-A and TNF-α treatment, respectively (52 ± 26.5 % and 57 ± 28.3 % vs control 100 %, Fig. 5b).

Disruption or shedding of the glycocalyx can also be assessed by measuring the release/loss of key components such as syndecan, heparan sulphate and hyaluronan. Syndecan-1 is a proteoglycan core protein while hyaluronan or hyaluronic acid (HA) is one of the glycosaminoglycans (GAG) and both contribute to the stability of the glycocalyx structure [23]. HA or syndecan-1 release into the supernatant during lung tumour CM exposure was used as an additional measure of glycocalyx degradation; determined respectively by hyaluronan and syndecan-1 ELISA. After 30 min, significantly increased levels of HA were detected in the SK-MES-1 CM treated endothelial supernate (56.94 ± 23.6 ng/ml vs control, 0.74 ± 0.04 ng/ml) and raised syndecan-1 levels (1.15 ± 1.09 ng/ml vs control [not detected]) were observed in the A549 CM exposed endothelial supernates (Fig. 5c).

## Discussion

Although, the exact mechanisms involved in the arrest and extravasation of CTCs during metastasis are still not fully understood, adhesion to, and transmigration through, the vascular endothelium is critically required. In the brain, the endothelium is an integral component of the blood–brain barrier (BBB), which due to its highly impermeable nature, presents a formidable challenge to the passage of metastasising CTCs.

An increasing number of reports highlight a role for endothelial adhesion molecules in the tethering of CTCs to the endothelium. This process may be additionally regulated by the steric effects of the endothelial glycocalyx which, in health, shields the adhesion molecules from circulating cells. Glycocalyx loss in response to metastasising tumour cells could favour attachment of tumour cells to the vessel wall, facilitating metastatic progression [24]. We hypothesised that both adenocarcinoma and squamous human lung tumour cells metastasising to the brain actually secrete factors that facilitate their own adhesion to the cerebral endothelium, thus enhancing their metastatic potential. Our data indicate, for the first time, that adenocarcinoma and squamous human lung tumour cells secrete proteins that not only disturb the integrity of the human cerebral endothelial glycocalyx, but concomitantly increase endothelial surface exposure of E-selectin and the binding of flowing tumour cells to the cerebral endothelium. Additionally, human lung adenocarcinoma tumour cells secrete proteins that enhance P-selectin exposure. These data suggest a critical role for the glycocalyx and cerebral endothelial adhesion molecules in the initial stages of extravasation during lung tumour metastasis to the brain.

Initial proteomic analysis of the secretome of two NSCLC subtypes, A549 (adenocarcinoma) and SK-MES-1 (squamous cell carcinoma), detected several secreted proteins with a potential role in tumour development or metastasis *i.e.* CC, CL, IGFBP-7, VEGF-A and TNF-α. It is interesting to note that the measured secreted levels of VEGF were relatively low compared to some of the other proteins detected. However, comparable, picogram levels of VEGF-A have been reported in the plasma of cancer patients [25] and to have significant effects on endothelial function [26]. The pathogenic role of VEGF in tumour growth is well documented (reviewed in [17]) and not only has enhanced VEGF expression been observed in all forms of NSCLC, but also high serum levels have been correlated with poor prognosis in patients [27, 28]. TNF-α, however, has been reported to have both pro- and anti-tumour effects [29]. For instance, in NSCLC a positive correlation has been identified between high TNF-α expression and favourable prognosis [30]. In contrast, not only have pro-tumourigenic effects of TNF-α been reported in various cancers *e.g.* breast [14], but interestingly, anti-TNF-α treatment of inflammatory diseases has led to a significant rise in the rates of malignancies in patients [31]. CC is a potent inhibitor of cysteine proteases. It also has contradictory roles in cancer pathogenesis; while expression of CC has been shown to be down-regulated in various aggressive metastatic tumours including prostate [32], increased plasma levels have been reported in patients suffering from a range of malignancies including lung cancer [16]. CL is an endopeptidase that degrades a range of proteins including collagen I and IV and elastin. It has been reported to promote cancer invasiveness by catalysing breakdown of the interstitial matrix and basement membrane [15]. IGFBP-7 is a member of the IGFBP superfamily [33] and binds to insulin-like growth factors (IGFs) with high affinity; regulating their activity by preventing ligand/receptor interactions. We and others have demonstrated a pro-angiogenic role for IGFBP-7. Exogenous IGFBP-7 induced proliferation, migration and tubule formation in omental microvascular endothelial cells [18] and induced tube-like structure formation in brain ECs [34]. Additionally, IGFBP-7 is implicated in the biology of a range of tumours including lung squamous cell carcinoma [35] and head and neck squamous cell carcinoma [36]. All five of these identified secreted-proteins and complete tumour CM significantly enhanced exposure of E-selectin on human cerebral microvascular endothelial monolayers and importantly, these changes were reflected in increased binding of the relevant lung tumour cells to the endothelial monolayer during 10 min perfusion.

The role of E-selectin in the lung tumour/endothelial adhesion was confirmed by blocking experiments using an anti-E-selectin antibody which completely attenuated the enhanced tumour/endothelial cell binding induced by the lung tumour secreted-factors. Additionally, flow cytometry analysis confirmed the expression of the E-selectin ligand sLeX on the surface of both lung tumour cell types. The observation that sLeX expression was higher in the A549 cell population than in SK-MES-1 cells is consistent with other studies that have demonstrated greater expression of sLeX antigens in lung adenocarcinomas than in squamous cell carcinomas [37]. The enzymes responsible for synthesizing sLeX such as α (1, 3) - fucosyltransferases are up-regulated in lung carcinomas, which could contribute to the adhesive and metastatic potential of lung cancer cells [38].

The observed role for E-selectin in lung tumour metastasis to the brain is in agreement with studies implicating E-selectin in metastasis in other cancer models. For instance, recent studies in a mouse model have demonstrated endothelial and tumour cell expression of E-selectin in brain metastases induced by metastatic murine mammary carcinoma cells [8]. The rapid (*i.e.* within 30 min) enhancement of lung tumour adhesion and surface exposure of E-selectin in both lung tumour cell types suggested that the tumour cells triggered additional adhesive mechanisms other than simply stimulating a transcriptionally regulated increase in surface expression of E-selectin. This raised the possibility that the interaction between the lung cancer cells and the endothelium is additionally regulated by the endothelial glycocalyx. Since, in health the length of glycocalyx components often exceeds that of the endothelial adhesion molecules, we investigated whether factors secreted from lung tumour cells could induce the rapid loss of cerebral endothelial glycocalyx; exposing the previously shielded adhesion molecules and facilitating adhesion. We show for the first time that factors secreted from both lung cancer subtypes rapidly and significantly induced degradation of the cerebral glycocalyx layer as assessed by WGA-FITC binding and shedding of glycocalyx components. Our previous data has confirmed that this lectin almost exclusively stains the luminal glycocalyx in cultured ECs [22]. The mechanism of glycocalyx modification or degradation in response to all of the tumour secreted-proteins can only be speculated at this point. These data are the first to show that CL, CC and IGFBP-7 induce glycocalyx changes and for the latter two proteins there is no available literature describing a role in glycocalyx degradation. CL may mediate glycocalyx damage indirectly by activating other glycocalyx degrading enzymes, *e.g.* heparanase, since formation of the active form of this enzyme from its precursor is exclusively catalysed by CL. However, since CL induced such rapid glycocalyx changes in cerebral endothelial cells an as yet unidentified, direct role on glycocalyx components cannot be ruled out.

TNF-α and VEGF have both been reported to alter the glycocalyx in other endothelial cell types. Henry and Duling suggested that TNF-α could directly stimulate the endothelium to either shed glycocalyx components, release free radicals, or activate surface-bound proteases or phospholipases that degrade it, such as endothelial-derived metalloproteases [39]. VEGF is able to directly regulate synthesis and shedding of glycocalyx components *e.g.* in glomerular endothelial cells [40] and has also been reported to induce changes in vessel permeability by causing structural glycocalyx alterations [41].

It is interesting to note that while significant and functionally relevant glycocalyx changes were observed within 30 min, the effects of longer treatment of cerebral endothelial cells with lung tumour secreted-factors induced even greater levels of E-selectin exposure and tumour cell adhesion. It is thus possible that the effects of lung tumour secreted factors is two-fold – not only inducing rapid glycocalyx perturbations which facilitate functional responses *i.e.* increased adhesion, over the shorter term, but inducing enhanced longer term responses resulting from even greater glycocalyx loss augmented by induction of increased cellular E-selectin protein expression and translocation to the cell surface.

## Conclusions

In summary, our data indicate a critical role for the cerebral EC glycocalyx and E-selectin/sLeX axis in regulating the adhesion of circulating lung tumour cells to the brain microvasculature; a key process in metastatic colonisation. We propose a cascade initiated by the secretion of pro-metastatic proteins from the lung tumour cells, which alter the cerebral endothelial glycocalyx structure revealing the E-selectin molecules on the endothelial cell surface. The E-selectin is then available to bind to sLeX ligand on the tumour cells facilitating the stronger bonds required for tethering and capture, ultimately enabling extravasation and secondary growth in the brain. Our data suggest that combining inhibitors capable of interrupting the contacts between E-selectin and its ligand along with preservation of glycocalyx integrity may represent a novel treatment modality for reducing brain metastasis in primary lung tumour patients.

## Additional files

Additional file 1:
**Supplementary methods (detailed description of MS Analysis, E-selectin ELISA, flow cytometry and legends for** **Additional file** **3:** **Figure S1 and Additional file** **4:** **Figure S2).** (DOCX 20 kb) Additional file 2:
**Supplementary tables S1, S2 and S3.** (DOCX 22 kb) Additional file 3: Figure S1. Supplementary cell adhesion figure depicting specificity of anti-E-selectin antibody. (PNG 234 kb) Additional file 4: Figure S2. Supplementary flow cytometry scatter plots for isotype-matched (IgM, κ) and positive controls. (PNG 722 kb)

## Abbreviations

EC: Endothelial cell(s)
CM: Conditioned-medium
IGFBP-7: Insulin-like growth factor-binding protein
ICAM-1: Intercellular adhesion molecule-1
NSCLC: Non-small cell lung cancer
sLeX: Sialyl lewis X
TNF-α: Tumour necrosis factor-alpha
VCAM-1: Vascular cell adhesion molecule-1
VEGF: Vascular endothelial growth factor
WGA-FITC: Wheat germ agglutinin conjugated to fluorescein isothiocyanate
