# Supplementary material for: Loss of the endothelial glycocalyx is associated with increased E-selectin mediated adhesion of lung tumour cells to the brain microvascular endothelium
Source: J Exp Clin Cancer Res. 2015 Sep 25;34:105. doi: 10.1186/s13046-015-0223-9 (PMC4582832; doi:10.1186/s13046-015-0223-9)
Supplement: Additional file 1: — Supplementary methods (detailed description of MS Analysis, E-selectin ELISA, flow cytometry and legends for Additional file 3: Figure S1 and Additional file 4: Figure S2). (DOCX 20 kb) [file 13046_2015_223_MOESM1_ESM.docx]

**Supplementary Methods**

**Mass Spectrometry Analysis**

LC-MALDI TOF/TOF mass spectrometry was carried out by Dr Heidi Fuller (The Robert Jones and Agnes Hunt Orthopaedic and District Hospital, Oswestry, UK). One millilitre of either A549 or SK-MES-1 CM or DMEM-BS was mixed with 10μl of StrataClean^TM^ Resin (Stratagene^®^). The mixture was vortexed and centrifuged for 1 minute at 2000rpm. The supernatant was removed and the pelleted samples were digested overnight at 30°C with 20μl of trypsin (20μg/ml, sequencing grade, Promega, Southampton, UK). Samples were then separated by liquid chromatography on an UltiMate^®^ 3000 Dionex system where they were loaded onto a C18 trapping column (10μl) and then eluted onto a C18 PepMap^TM^ analytical column. Peptides were eluted using a 40 minute acetonitrile (MeCN) gradient (2%-50% MeCN/ H_2_O) followed by a further elution at 90% MeCN for 10 minutes. The column was washed and equilibrated for 10 minutes. Samples were spotted at 10 second intervals using a Probot^TM^ Microfraction Collector with alpha-cyano-4-hydroxycinnamic acid (α-CHCA, 3mg/ml in 70% MeCN/ 0.1% TFA) at a continuous flow rate of 1.2μl/min. Sample plates were then placed in a 4800 MALDI TOF/TOF^TM^ Analyser (Applied Biosystems, Warrington, UK) for mass spectrophotometry and close external standards were used to calibrate the instrument. Proteins were identified using Peak Explorer^TM^ software. Identified proteins with a total ion score (Confidence Interval, C.I) of >95%, with two or more identified peptides, were deemed as true interactions. However, any proteins with lower scores could indeed be true if their presence could be confirmed by further biochemical methods. Total ion scores were calculated from weighted ion scores for individual peptides that were matched to a given protein.

**Cell surface based E- and P-selectin ELISA**

Following treatment, hCMEC/D3 cells were washed with DMEM-BS and incubated with mouse anti-human antibody against E- or P-selectin (5µg/ml in PBS-1% BSA, 50µl per well) for 1 hour at 4^o^C. Following three washes with PBS-BSA, a biotinylated goat anti-mouse secondary antibody (0.64µg/ml in PBS-BSA, 50µl per well) (Sigma Aldrich, Gillingham, UK) was applied to the wells for another hour at 4^o^C. After washing as above, the cells were incubated with a solution of streptavidin alkaline phosphatase (2µg/ml in Tris buffered saline, 100µl per well) for 30 minutes at 4^o^C, washed and incubated with para-nitrophenyl phosphate (*p-NPP*) substrate solution for 30 minutes in the dark. The enzyme reaction was stopped with 3M NaOH prior to reading the absorbance on a plate reader (BMG Labtech FLUOstar OPTIMA, BMG LABTECH Ltd. Aylesbury, UK) at 405nm.

**Flow cytometry analysis of adhesion molecule counter ligands/receptors**

Lung tumour cells at approximately 80% confluence were detached using non-enzymatic cell dissociation solution (Sigma Aldrich, Gillingham, UK) and centrifuged (600*g*, 5 minutes) before incubation with 10% (v/v) blocking serum (Sigma Aldrich, Gillingham, UK) for 30 minutes at room temperature (RT). After centrifugation (600*g*, 10 minutes), A549 and SK-MES-1 cells were incubated with 1µg mouse anti-human primary antibodies against CD49d, CD11a, CD11b, P-selectin glycoprotein ligand-1 (PSGL-1) (all ImmunoTools, Germany) and sialyl Lewis X (sLeX, BD Biosciences, Oxford, UK) for 30 minutes on ice. The cells were then washed in PBS prior to incubation with anti-mouse secondary antibody conjugated to FITC (1:300) (Sigma Aldrich, Gillingham, UK) for 30 minutes in the dark at RT. Following washes, samples (in 500µl PBS) were analysed on a Beckman Coulter Cell Lab Quanta SC flow cytometer and data for 10,000 events were collected. The percentage of positively stained cells was identified and the results were recorded. Cells stained with relevant isotype control primary antibody (IgM, κ) were subtracted from positive cell staining with test antibody to control for test antibody specificity and avoidance of false-positive results.

**Hyaluronan Quantikine® Immunoassay and Syndecan-1 ELISA**

Fully confluent hCMEC/D3 cells, grown in 12 well plates, were treated for 30 minutes with either DMEM-BS or freshly prepared A549 or SK-MES-1 CM at 37^o^C. The supernatants (500µl), after centrifugation at 600*g* for 10 minutes and 4^o^C, were collected and analysed immediately using the Hyaluronan Quantikine ELISA kit (R&D Systems, Abingdon, UK) or the Syndecan-1 (CD138) ELISA kit (Abcam, Cambridge, UK) according to the manufacturer’s instructions. Preliminary experiments were performed to determine optimum dilution requirements. In order to determine the exact amount of hyaluronan being released from the brain endothelial glycocalyx, HA and syndecan-1levels were also measured in both A549 and SK-MES-1 CM alone. The latter was then deducted from the hCMEC/D3 supernate values to give the final concentrations.

**Figure S1** Effects of IgG_1_ isotype control antibody on the adhesion of A549 and SK-MES-1 cells to TNF-α stimulated hCMEC/D3 monolayers. Here, confluent brain endothelial monolayers were treated with 160pg/ml TNF-α for 24hrs prior to incubation with 5µg/ml mouse monoclonal IgG_1_ antibody for 30minutes at 37^o^C. Thereafter, hCMEC/D3 monolayers were perfused with Calcein AM-stained A549 or SK-MES-1 cells for 10minutes. Exposure of the isotype-matched antibody had no significant effects on the increased adhesion of A549 and SK-MES-1 cells induced by TNF-α.

**Figure S2** Isotype-matched (IgM, κ) and positive controls for flow cytometry analysis of adhesion molecule ligands/receptors. The EV-SS plot along with their corresponding histogram and tables obtained for the isotype control antibody indicate that the positive staining observed during sialyl Lewis X expression studies was not due to any non-specific binding of the primary antibody in both A549 **(a)** and SK-MES-1 **(b)** lung tumour cells. Expression profiles of CD11a, CD49d and CD11b in U937, a monocytic cell line, and freshly isolated monocytes (positive controls). U937 cells were incubated with 1μg CD11a **(c)** and CD49d **(d)** primary antibodies and FITC-conjugated secondary antibody prior to expression analysis by flow cytometry. However, U937 displayed very low levels of CD11b and as such freshly isolated monocytes were used as a positive control for this antigen **(e)**.
