# Supplementary material for: Loss of the endothelial glycocalyx is associated with increased E-selectin mediated adhesion of lung tumour cells to the brain microvascular endothelium
Source: J Exp Clin Cancer Res. 2015 Sep 25;34:105. doi: 10.1186/s13046-015-0223-9 (PMC4582832; doi:10.1186/s13046-015-0223-9)
Supplement: Additional file 2: — Supplementary tables S1, S2 and S3. (DOCX 22 kb) [file 13046_2015_223_MOESM2_ESM.docx]

| **Rank** | **Protein Name** | **Accession Number** | **Peptide Count** | **Total Ion Score C.I. %** |
| --- | --- | --- | --- | --- |
| 1 | Transferrin [Homo sapiens] | gi\|37747855 | 9 | 100 |
| 2 | fibronectin precursor [Homo sapiens] | gi\|31397 | 9 | 100 |
| 3 | actin, beta [Homo sapiens] | gi\|14250401 | 8 | 100 |
| 4 | glucose-6-phosphate dehydrogenase [Homo sapiens] | gi\|452269 | 6 | 99.99951 |
| 5 | nucleobindin 1 [Homo sapiens] | gi\|20070228 | 6 | 99.99811 |
| 6 | cathepsin L1 preproprotein  [Homo sapiens] | gi\|4503155 | 4 | 99.99753 |
| 7 | beta amyloid peptide precursor | gi\|226343 | 5 | 99.99565 |
| 8 | Chain A, Human Cystatin C; dimeric form With 3D domain Swapping | gi\|14278690 | 4 | 99.92613 |
| 9 | aldo-keto reductase family 1, member B10 [Homo sapiens] | gi\|20127592 | 4 | 99.90828 |
| 10 | FLJ00343 protein [Homo sapiens] | gi\|21748542 | 6 | 99.88059 |
| 11 | enolase 1 [Homo sapiens] | gi\|4503571 | 2 | 99.65686 |
| 12 | Heterogeneous nuclear ribonucleo- protein A2/B1 isoform B1  [Homo sapiens] | gi\|14043072 | 3 | 99.18628 |
| 13 | NUANCE [Homo sapiens] | gi\|17016967 | 6 | 99.05587 |
| 14 | Heterogeneous nuclear ribonucleo protein A1 (Helix-destabilizing protein) (Single-strand RNA-binding) | gi\|133254 | 1 | 99.01943 |
| 15 | insulin-like growth factor binding protein 7 [Homo sapiens] | gi\|4504619 | 2 | 98.71626 |
| 16 | cingulin [Homo sapiens] | gi\|16262452 | 3 | 98.55297 |
| 17 | aldo-keto reductase family 1, member D1 [Homo sapiens] | gi\|5174695 | 2 | 98.3038 |
| 18 | PREDICTED: cathepsin H isoform 6 [Pan troglodytes] | gi\|114658412 | 2 | 98.26029 |
| 19 | BIGH3 [Homo sapiens] | gi\|2996636 | 2 | 97.89391 |
| 20 | dihydrodiol dehydrogenase isoform DD 1 [Homo sapiens] | gi\|556516 | 2 | 97.26803 |
| 21 | laminin alpha5 chain precursor  [Homo sapiens] | gi\|20147503 | 4 | 96.042 |
| 22 | Titin [Homo sapiens] | gi\|17066105 | 11 | 95.33925 |

**Table S1 Proteins identified in the A549 CM sample.** The protein rank, peptide count and total ion score C.I% are shown.

| **Rank** | **Protein Name** | **Accession Number** | **Peptide Count** | **Total Ion Score C.I. %** |
| --- | --- | --- | --- | --- |
| 1 | Transferrin [Homo sapiens] | gi\|37747855 | 6 | 100 |
| 2 | Chain A, Crystal Structure Of Human Serum Albumin | gi\|3212456 | 2 | 100 |
| 3 | Insulin-like growth factor binding protein 7  [Homo sapiens] | gi\|4504619 | 4 | 100 |
| 4 | Beta actin [Homo sapiens] | gi\|4501885 | 5 | 100 |
| 5 | Heat shock 90kDa protein 1, beta [Homo sapiens] | gi\|20149594 | 5 | 100 |
| 6 | Prosaposin isoform b prepro protein [Homo sapiens] | gi\|110224476 | 2 | 99.99984051 |
| 7 | FLJ00343 protein  [Homo sapiens] | gi\|21748542 | 5 | 99.99680322 |
| 8 | Unnamed protein product [Homo sapiens] | gi\|16552261 | 3 | 99.99665257 |
| 9 | Glucose-6-phosphate dehydrogenase [Homo sapiens] | gi\|452269 | 4 | 99.99198854 |
| 10 | Alpha glucosidase II alpha subunit isoform 2  [Homo sapiens] | gi\|38202257 | 4 | 99.98806804 |
| 11 | Transforming growth factor, beta-induced, 68kDa  [Homo sapiens] | gi\|4507467 | 4 | 99.96470515 |
| 12 | Histone cluster 1, H2ae  [Homo sapiens] | gi\|10645195 | 2 | 99.95536132 |
| 13 | Unnamed protein product [Homo sapiens] | gi\|31092 | 3 | 99.95127953 |
| 14 | heat shock protein 90kDa alpha (cytosolic), class A member 1 isoform 2 [Homo sapiens] | gi\|154146191 | 3 | 99.81132624 |
| 15 | Fibronectin precursor  [Homo sapiens] | gi\|31397 | 3 | 99.80197910 |
| 16 | Tubulin, alpha 1B [Mus musculus] | gi\|34740335 | 1 | 99.43543841 |
| 17 | Sparc/osteonectin, cwcv and kazal-like domains proteo glycan (testican) 1  [Homo sapiens] | gi\|21265163 | 2 | 99.42758439 |
| 18 | Heat shock 70kDa protein 8 isoform 1 [Homo sapiens] | gi\|5729877 | 2 | 99.40198550 |
| 19 | Collagen, type VI, alpha 1 precursor [Homo sapiens] | gi\|87196339 | 3 | 99.33822373 |
| 20 | Chain A, Human Cystatin C; Dimeric Form With 3d Domain Swapping | gi\|14278690 | 2 | 99.24714502 |
| 21 | Heterogeneous nuclear ribo nucleoprotein A2/B1 isoform B1 [Homo sapiens] | gi\|14043072 | 3 | 99.16109757 |
| 22 | N2B-Titin Isoform  [Homo sapiens] | gi\|17066104 | 8 | 98.85694535 |
| 23 | Unnamed protein product [Homo sapiens] | gi\|7020225 | 3 | 98.71556746 |
| 24 | Titin [Homo sapiens] | gi\|17066105 | 11 | 98.61296195 |
| 25 | Heat shock protein 70 testis variant [Homo sapiens] | gi\|3461866 | 2 | 98.60455938 |
| 26 | Alpha2-HS glycoprotein [Homo sapiens] | gi\|2521981 | 1 | 98.24728410 |
| 27 | Cytosolic thyroid hormone-binding protein (EC 2.7.1.40) | gi\|338827 | 4 | 97.80359977 |
| 28 | Nebulin | gi\|19856971 | 2 | 97.60521144 |
| 29 | Golgi antigen gcp372  [Homo sapiens] | gi\|808869 | 4 | 97.50201880 |
| 30 | Histone H4 | gi\|223582 | 1 | 97.20931261 |
| 31 | Putative [Homo sapiens] | gi\|553734 | 1 | 96.08518438 |
| 32 | KIAA0841 protein  [Homo sapiens] | gi\|33869699 | 3 | 93.72359101 |
| 33 | Acyl-Coenzyme A dehydroge nase, very long chain isoform 1 precursor [Homo sapiens] | gi\|4557235 | 3 | 92.47145020 |
| 34 | Protein kinase C substrate 80K-H isoform 1  [Homo sapiens] | gi\|48255889 | 3 | 92.11664266 |
| 35 | Aldolase A [Homo sapiens] | gi\|4557305 | 4 | 91.29615 |

**Table S2 Proteins identified in the SK-MES-1 CM sample.** The protein rank, peptide count and total ion score C.I% are shown.

| **Rank** | **Protein Name** | **Accession Number** | **Peptide Count** | **Total Ion Score C.I. %** |
| --- | --- | --- | --- | --- |
| 1 | Transferrin [Homo sapiens] | gi\|37747855 | 8 | 100 |
| 2 | TGF-beta resistance-associated protein TRAG [Homo sapiens] | gi\|15624075 | 1 | 98.62989076 |
| 3 | human type 3 inositol 1,4,5-trisphosphate receptor | gi\|393036 | 3 | 98.25516197 |
| 4 | hypothetical protein [Homo sapiens] | gi\|5262501 | 2 | 97.51826734 |
| 5 | centromere protein F [Homo sapiens] | gi\|55770834 | 4 | 95.93681220 |
| 6 | restin isoform a [Homo sapiens] | gi\|4506751 | 2 | 95.43169441 |

**Table S3 Proteins identified in DMEM-BS sample**. The protein rank, peptide count and total ion score C.I% are shown.

| **Components** | **Contents g/L** |
| --- | --- |
| Sodium Chloride | 6.4 |
| Potassium Chloride | 0.4 |
| Calcium Chloride 2H_2_0 | 0.264 |
| Magnesium Sulphate 7H_2_0 | 0.2 |
| Sodium Dihydrous Phosphate 2H_2_0 | 0.141 |
| Dextrous Anhydrous | 1 |
| Ferric Nitrate 9H20 | 0.0001 |
| L-Glutamine | 0.0116 |
| Sodium Pyruvate | 0.0022 |
| Phenol Red | 0.0003 |
| Sodium Bicarbonate | 0.074 |
| L-Arginine-HCl | 0.084 |
| L-Histidine-HCl-H_2_0 | 0.042 |
| L-Isoleucine | 0.105 |
| L-Leucine | 0.105 |
| L-Lysine-HCl | 0.146 |
| L-Methionine | 0.03 |
| L-Phenylalanine | 0.066 |
| L-Threonine | 0.095 |
| L-Tryptophan | 0.016 |
| L-Valine | 0.094 |
| Glycine | 0.03 |
| L-Serine | 0.042 |
| L-Cystine | 0.048 |
| L-Tyrosine | 3.6 |
| Choline-Chloride | 0.004 |
| Nicotinamide | 0.004 |
| D-Calcium Pantothenate | 0.004 |
| Pyridoxine-HCl | 0.004 |
| Thiamine-HCl | 0.004 |
| Riboflavin | 0.0004 |
| Folic Acid | 0.004 |
| I-Inositol | 0.0072 |
| Sodium Selenite | 0.0000346 |
| Putrescine | 0.0145 |
| Tri-Iodo L Thyronine | 0.000303 |
| Progesterone | 0.0000566 |
| L-Thyroxine | 0.00036 |
| Insulin (Human Recombinant) | 0.001 |
| Human Transferrin | 0.1 |

**Table B Composition of Dulbecco’s Modified Eagles Medium-Bottenstein Sato (DMEM-BS)**
