# Supplementary figures and images for: Loss of the endothelial glycocalyx is associated with increased E-selectin mediated adhesion of lung tumour cells to the brain microvascular endothelium
Source: J Exp Clin Cancer Res. 2015 Sep 25;34:105. doi: 10.1186/s13046-015-0223-9 (PMC4582832; doi:10.1186/s13046-015-0223-9)

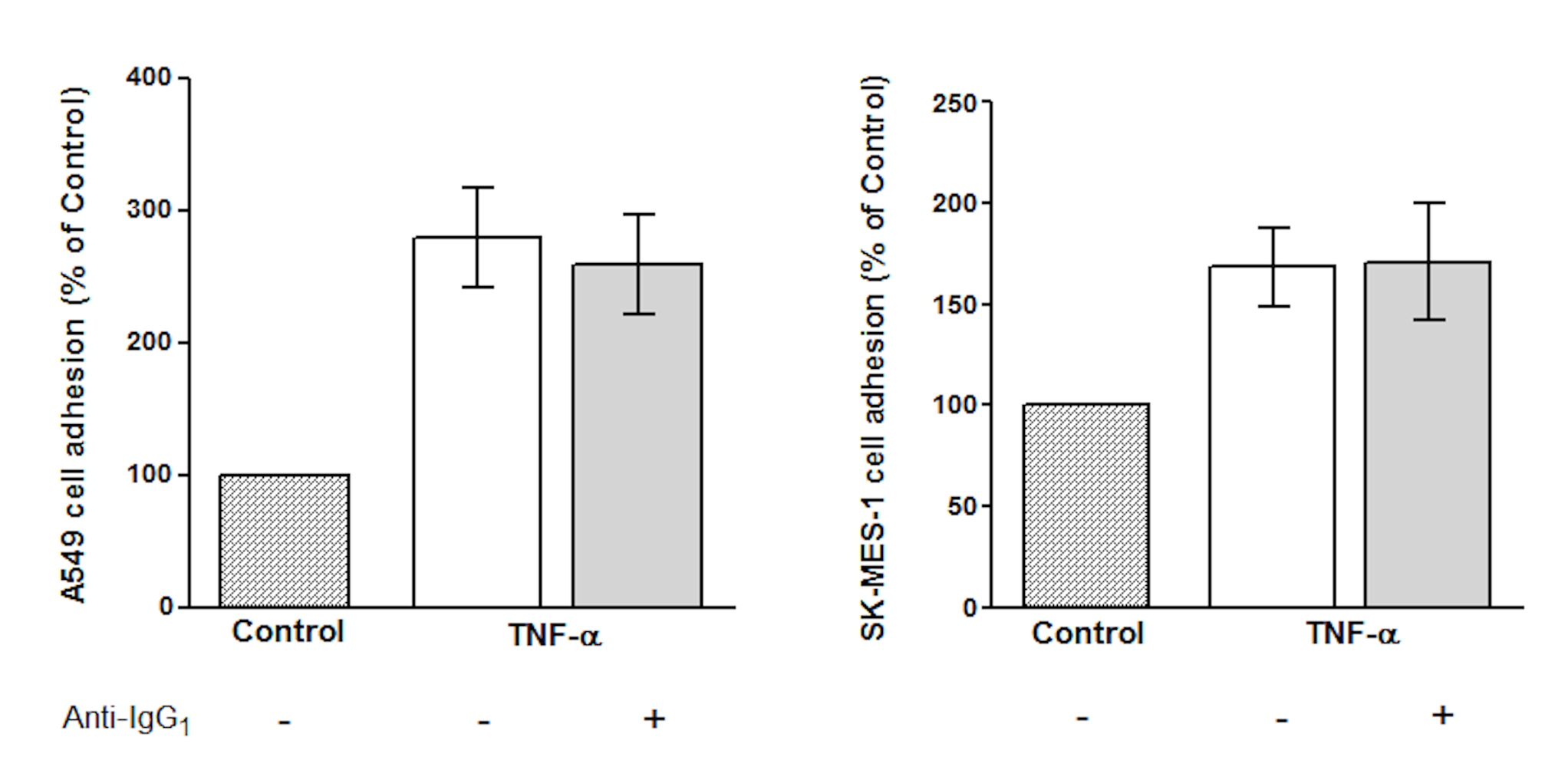

Supplement: Additional file 3: Figure S1. — Supplementary cell adhesion figure depicting specificity of anti-E-selectin antibody. (PNG 234 kb) [file 13046_2015_223_MOESM3_ESM.png]

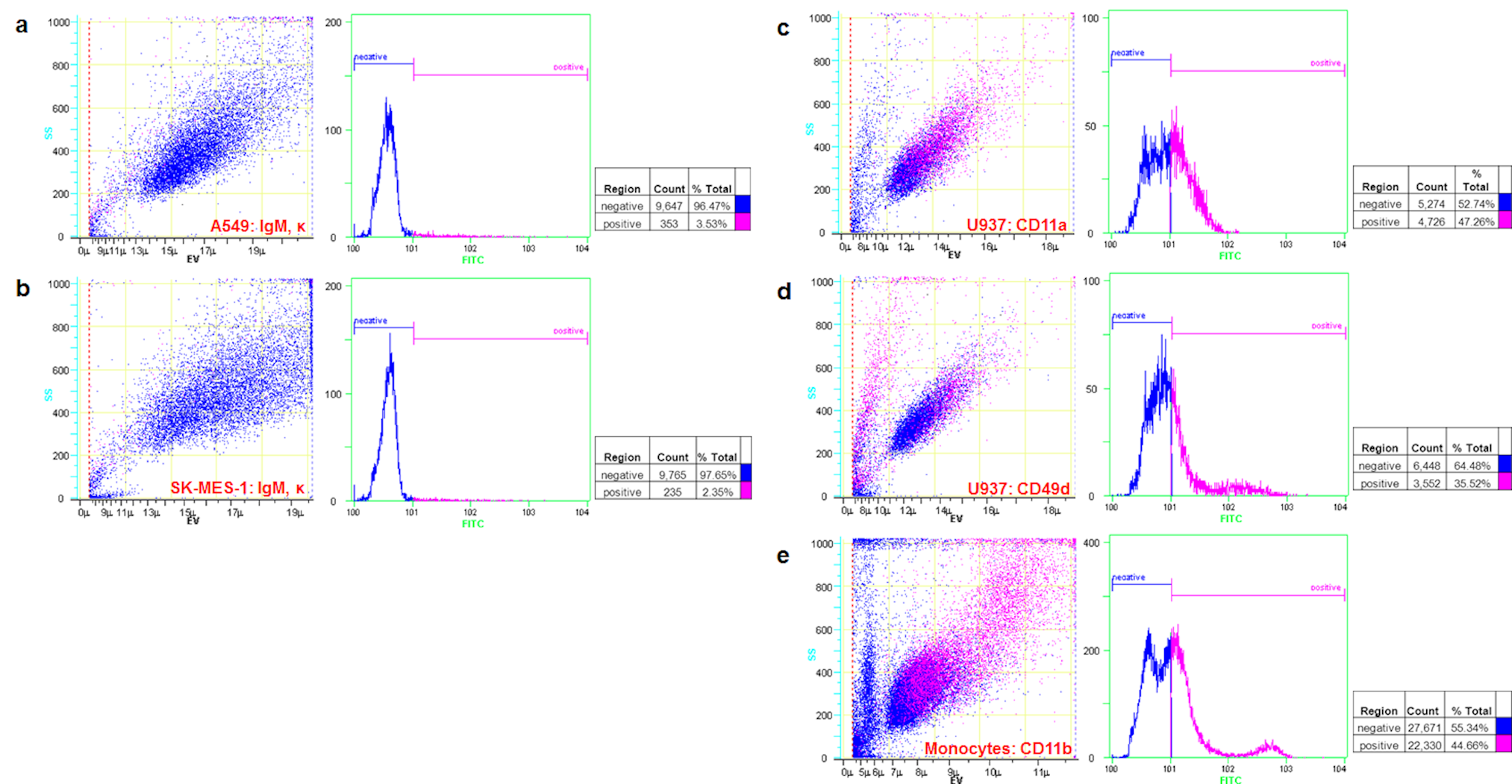

Supplement: Additional file 4: Figure S2. — Supplementary flow cytometry scatter plots for isotype-matched (IgM, κ) and positive controls. (PNG 722 kb) [file 13046_2015_223_MOESM4_ESM.png]
